# OA04.02. Mechanisms of growth inhibition of pancreatic cancer by omega-3 polyunsaturated fatty acids

**DOI:** 10.1186/1472-6882-12-S1-O14

**Published:** 2012-06-12

**Authors:** M Chen, V Go, O Hines, G Eibl

**Affiliations:** 1 David Geffen School of Medicine at UCLA, Department of Surgery, Los Angeles, USA

## Purpose

Omega-3 polyunsaturated fatty acids (PUFAs) are widely considered health promoting. We have previously reported that the omega-3 PUFA eicosapentaenoic acid (EPA) inhibits growth of pancreatic cancer (PaCa) cells *in vitro* and *in vivo*. However, the mechanism underlying the effects of EPA in PaCa cells is unknown.

## Methods

Six human PaCa cell lines of varying degrees of differentiation were exposed to different concentrations and times to EPA. Cell growth was measured by BrdU and apoptosis was determined by a Cell Death ELISA, and cleavage of PARP and caspase-3/7. The involvement of PI3K/Akt, a major survival pathway in PaCa, was determined using pharmacological inhibitors (LY294002) and constitutively active Akt (myr-Akt1). The effect of EPA on *de novo* fatty acid synthesis as a downstream pathway of Akt inhibition was investigated using Western blotting and inhibitors of fatty acid synthase (FASN).

## Results

EPA dose-dependently increased apoptosis and stimulated cleavage of PARP and caspase-3/7, which was accompanied by inhibition of Akt. Exposure of PaCa cells to LY294002 induced apoptosis and decreased cell growth. EPA-induced apoptosis and growth inhibition was attenuated in myr-Akt1 transfected cells. FASN was expressed in all cell lines. Inhibition of FASN by C75, a synthetic FASN inhibitor, reduced cell growth and induced apoptosis. EPA inhibited FASN expression, which was accompanied by inhibition of cell growth. Insulin stimulated Akt phosphorylation and increased FASN expression, while LY294002 reduced FASN. PaCa cells transfected with myr-Akt1 exhibited increased FASN expression, indicating the importance of Akt in baseline FASN expression. Importantly, EPA decreased Akt phosphorylation and FASN expression in control-transfected cells, but this inhibition was abrogated in myr-Akt1 transfected cells.

## Conclusion

Our studies provide compelling evidence that the growth inhibitory effects of the omega-3 polyunsaturated fatty acid EPA in PaCa cells was mediated by inhibition of the PI3K/Akt pathway and subsequently reduced fatty acid synthesis.

